# Efficacy of Short Message Service Text Messaging Interventions for Postoperative Pain Management: Systematic Review

**DOI:** 10.2196/20199

**Published:** 2021-06-16

**Authors:** Christoph Buck, Christian Keweloh, Adam Bouras, Eduardo J Simoes

**Affiliations:** 1 School of Business and Economics Philipps-Universität Marburg Marburg Germany; 2 Project Group Business and Information Systems Engineering Fraunhofer Institute for Applied Information Technology FIT Bayreuth Germany; 3 UNITY AG Büren Germany; 4 School of Medicine University of Missouri Columbia, MO United States

**Keywords:** systematic literature review, pain management, opioid, short message service (SMS), postoperative

## Abstract

**Background:**

Addiction to opiates and synthetic opioids poses a major threat to public health worldwide, with pharmaceutical opioids prescribed to manage pain constituting the main problem. To counteract this threat, suitable pain management strategies should be implemented in health care. Monitoring pain management seems to be feasible using telemedicine with a certain degree of resource intensity and digitization. As a communication channel for this type of monitoring, SMS appears to be a valid alternative.

**Objective:**

The aim of this systematic literature review was to (1) provide information on the state of research regarding postoperative pain management via SMS, (2) establish a basic understanding of SMS-based pain management, and (3) provide insight into the feasibility of these management strategies. The research question was as follows: Is postoperative pain management feasible and effective utilizing SMS?

**Methods:**

A systematic literature review was performed mainly following the PRISMA guidelines and another guide on performing a systematic literature review for information systems–related research. A search string was developed based on the objectives and research question, and eight databases were searched.

**Results:**

The initial search resulted in 2083 records, which could be narrowed down by applying various exclusion criteria. Thereby, 11 articles were identified as relevant, which were accordingly analyzed and evaluated by full-text screening. In all articles, pain management interventions were performed using SMS communication between health care professionals and patients or their legal guardians. A prospective approach was predominantly chosen as the study design (91%) with the leading research objective of determining the intervention’s feasibility (73%). The primary reason for sending SMS messages was to monitor patients (64%). Overall, the use of SMS improved adherence, acceptance, and satisfaction regarding postoperative pain management. With an average response rate of approximately 89.5% (SD 3.8%), the reliability of SMS as a communication and monitoring tool was further emphasized. This response rate is significantly higher than that for email interventions (66.63%, *P*<.001).

**Conclusions:**

This study provides a comprehensive picture of the current status on postoperative pain management by SMS. Communication via SMS was beneficial in all interventions, even preoperative. Six SMS interventions could be certified by the respective institutional review board and three were Health Insurance Portability and Accountability Act–compliant. Therefore, the results of this study could be leveraged to address the opioid epidemic. Overall, the research question could be confirmed. Future research should extend this systematic literature review regarding preoperative pain management. Based on these findings, a pre- and postoperative communication model should be developed to address the opioid epidemic effectively.

## Introduction

### Background

Globally, the increasing use of opiates and synthetic opioids poses a major threat to public health [1,2]. In 2017, approximately 53 million people took opioids at least once in the past year, with the highest prevalence of nonmedical opioid use estimated in North America [2]. More than 700,000 people died from drug overdoses in the United States between 1999 and 2017, and approximately two-thirds of these cases involved opioids [3,4]. The leading causes of this epidemic are opioid misuse, an overall increase in opioid prescriptions, shifted patient expectations, inadequate medical education and practice, insufficient guidelines, and the highly addictive nature of opioids [1,3-8]. The major opioids of concern remain pharmaceutical opioids used for pain control [1,2], which are typically prescribed for postoperative pain management [9]. Therefore, suitable pain management strategies need to be developed and implemented to adequately address and counteract this opioid epidemic [10-12].

Almost every aspect of these pain management strategies, and the efficiency and quality of health care rely on effective communication [13,14]. Poor health care professional–patient interactions can lead to adverse clinical outcomes, insufficient patient understanding, poor patient compliance, and consequently negative outcomes [15,16]. Improving communication and implementing postoperative monitoring appears to be a practical approach, since less than half of patients report adequate postoperative pain relief [17]. However, resource-intensive pain management is difficult to implement in the health care sector due to the constant and increasing pressure to provide patient care most efficiently and as cost-effectively as possible [18]. These demands could be fulfilled by increasing telemedicine interventions and measures [19-21].

Mobile health, delivered through mobile instant messaging apps or SMS texting, has particular potential in this regard. SMS is utilized more frequently [22], as SMS communication provides various features and proven benefits for health care applications. Unlike mobile instant messaging apps, no smartphone or internet access is required for SMS [22-24]. In the United States, 96% of the population already own a mobile phone and 97% of smartphone users have sent at least one SMS message within the week. With approximately 6 billion SMS messages sent daily, it is the most popular and widely used communication feature [25,26]. Furthermore, SMS is a low-cost, provider-independent, scalable, ubiquitous, reliable, secure, widely accepted, and simple communication means [22,23,26,27].

### Objective and Structure of the Study

To address the ongoing opioid epidemic, pain management combined with SMS as a communication medium appears to be very viable, whereby the postoperative phase seems to be unusually decisive. Therefore, the aim of this study was to structure the current state of the literature regarding postoperative pain management via SMS. To our knowledge, there is currently no specific literature review on SMS-based pain management and no synthesized results. Accordingly, this study examined the following research question: Is postoperative pain management feasible and effective utilizing SMS? By answering the research question through a systematic literature review, a conceptual framework for future research is provided.

To gain a valid answer to the research question, the paper is structured as follows. In the Methods, we describe the process of performing the systematic literature review, along with a detailed description of the specific selection and exclusion criteria. The results of the selected literature are compiled accordingly in the Results. The Discussion explains the principal insights from the included studies, along with the limitations of this review. Finally, we provide recommendations for action based on the conclusions, and highlight research gaps for researchers, clinicians, and other health care professionals.

## Methods

### Design

The goal of this literature review was to provide comprehensive insight into postoperative management via SMS. The review should (1) provide information on the state of research, (2) establish a basic understanding of SMS-based pain management, and (3) provide deep insight into the feasibility of these management strategies. To ensure completeness and transparency, a systematic literature review process was followed in all stages of the study. The methodology is mainly based on the PRISMA (Preferred Reporting Items for Systematic Reviews and Meta-Analyses) guidelines [28] and a guide for systematic literature reviews in information systems research [29]. A detailed and well-structured protocol was initially drafted, which fully defines the procedures to ensure validity and accuracy [28,30]. The protocol can be found in Multimedia Appendix 1.

### Search Strategy

**Figure 1:**
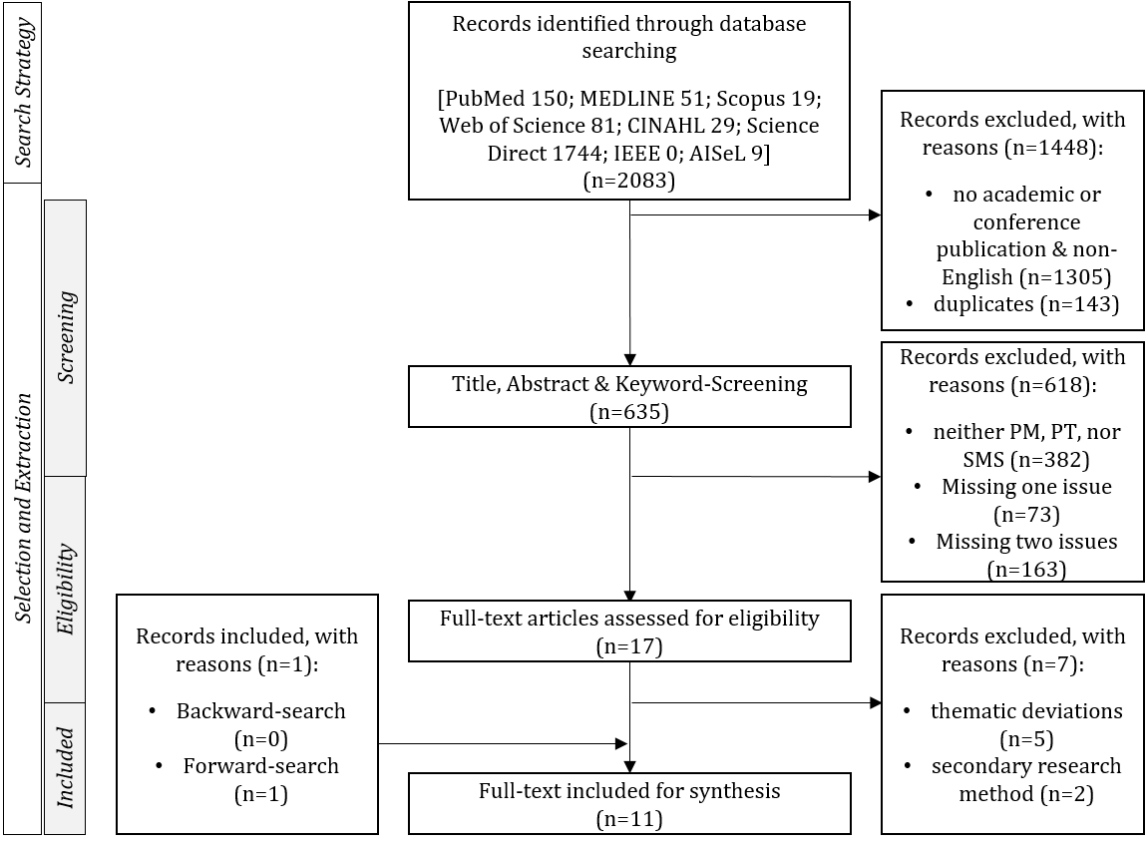
Systematic literature review flowchart. PM: pain management; PT: postoperative treatment.

In consideration of the research question, a search string was designed using Boolean operators (AND, OR) for the selection of relevant literature. Various keywords connected the decisive aspects regarding text messages, postoperative care, and pain management. The conclusive search string is documented in the protocol (Multimedia Appendix 1) and is composed in the detailed form of “text messag*” OR “short messag*” OR “sms” AND “postoperative care*” OR “postoperative care*” OR “surge*” OR “surgic*” OR “operat*” AND “pain” OR “medicat*” OR “opioid*” OR “analgesic.” The searches were performed in three databases in the medical field (PubMed, Medline, and CINAHL), three interdisciplinary databases (Web of Science, Scopus, and Science Direct), and two databases covering the field of computer science (IEEE and AISeL) (Figure 1). The search string was adapted to the specific characteristics and requirements of the respective database. The search was performed between March and April 2019.

### Selection and Extraction

The initial search in the eight databases resulted in 2083 matches. The screening process narrowed down this result by applying a variety of exclusion criteria. Initially, only articles that were published in academic journals or conferences were considered. Further, all non-English articles were excluded. By applying these two criteria, 1305 articles were already excluded. Next, all duplicates were identified by DOI or title alignment and deleted for the next steps (n=143). The remaining 635 articles were screened by title, abstract, and keywords for their relevance to the research question. Accordingly, the papers needed to describe and analyze pain management, and to clearly emphasize postoperative care and SMS. Articles that interpreted abbreviations such as SMS differently or focused on issues not relevant to the review were further excluded. During this screening process, a total of 382 articles could be identified as irrelevant, as these articles neither focused on pain management, postoperative treatment, nor SMS. Only two of these three issues were addressed in 73 articles and one of the three was addressed in 163 articles. As a result of this process, 618 articles were classified as irrelevant, leaving a total of 17 articles eligible for further review.

Full-text screening was performed to comprehensively analyze the remaining 17 articles, and an additional 7 articles were excluded due to thematic deviations or the secondary research method. Based on the remaining 10 articles, a forward and backward search was executed. The backward search revealed no new articles. The forward search resulted in a total of 64 matches for the eight databases. After applying the exclusion criteria, one additional article could be included for analysis, leaving 11 articles in the final pool for review (Figure 1) [28,31].

## Results

### Characteristics of Selected Studies

Of the 635 articles analyzed in the screening process, 554 (87.2%) were published in the last 5 years, since 2014. This reflects the increasing relevance of digitalized postoperative pain management in recent years. Correspondingly, this is apparent in the final pool of 11 articles, with 45% (n=5) published in 2018 and 36% (n=4) published in 2019. Three articles were published in the journal *Telemedicine and e-Health* [32-34], representing the interface between health and informatics. The remaining eight articles were published in various health-related journals. The average 2018 impact factor of the journals was 2.51, with the best-ranked journal at 6.03.

Ten of these 11 (91%) studies adopted a prospective design to investigate postoperative pain management in conjunction with SMS. Patients were grouped as cohorts, and investigated for pain and similar outcomes. One study was a nonblinded randomized control trial [35]. The main research aim of 8 of the 11 (73%) studies was to determine the intervention’s feasibility (Table 1).

**Table 1:** Characteristics of the 11 studies.

Reference	Study design	Research aim	Automation	Age (years), mean (SD)	Surgical procedure
Anthony et al [32]	Prospective multicenter cohort	Feasibility	yes	49.6 (13.7)	Hand surgery
Anthony et al [33]	Prospective cohort	Feasibility	yes	46.0 (22.0)	Lower extremity fracture
Booth et al [36]	Prospective cohort	Feasibility	n/a^a^	30.7 (5.5)	Caesarean section
Brix et al [35]	Nonblinded randomized control trial	Feasibility	yes	47.5 (16.5)	Knee arthroplasty
Carrier et al [37]	Prospective multicenter cohort	Validation	yes	57.0 (n/a)	Colorectal surgery
Chen et al [38]	Prospective cohort	Feasibility	yes	8.5 (n/a)	Tonsillectomy
Day et al [39]	Prospective cohort	Feasibility	yes	n/a	Total hip or knee arthroplasty
Nelson et al [40]	Prospective cohort	Feasibility	yes	6.1 (2.1)	Humeral fractures
Newton and Sulman [41]	Prospective cohort	Feasibility	n/a	n/a	Tonsillectomy
Premkumar et al [42]	Prospective cohort	Validation	yes	59.4 (10.9)	Total hip or knee arthroplasty
Yahanda et al [34]	Prospective cohort	Validation	yes	n/a	Total hip or knee arthroplasty

^a^ n/a: not available; the article did not provide corresponding information.

### Characteristics of Study Populations

Overall, 4195 patients were supported by a pain management system tailored to the surgery performed and to the patients’ characteristics such as age or physical condition. The number of study participants ranged from 21 to 3049, with a mean of 381 participants and a median of 85. To select these patients, the researchers applied various selection criteria. For instance, 9 of the 11 (82%) research teams excluded patients without a mobile phone, 6 (55%) excluded patients who could not communicate via SMS, and 5 (45%) excluded patients with a language barrier. At the beginning of the intervention, the sex and age, and other demographic data of all participants were determined in 8 of 11 (73%) studies. The percentage of female participants ranged between 33% and 100%. The intervention was directed at adult patients with a mean age between 30.7 and 59.4 years in 6 of 8 (75%) studies. The other 2 studies analyzed pain management in children between 6.1 and 8.2 years of age (Table 1). The educational level and work status were determined in 4 of these 8 (50%) studies. The percentage of participants with an educational level above a bachelor's degree varied between 23% and 43%, and the proportion of participants with full-time employment ranged between 40% and 75%. The race, BMI, and surgical history of the patients were identified in 3 of the 8 (38%) studies. Six of the 11 (55%) studies carried out a software demonstration.

### Intervention Characteristics of Selected Studies

Nine of the 11 studies used automated pain management systems for their interventions and the other 2 studies did not provide any relevant information on this aspect [36,41]. For 7 of the 11 (64%) studies, the primary reason for sending text messages was to monitor patients’ postoperative pain. The remaining 4 (36%) studies intended to determine adherence to the pain treatment, which was combined with a teaching purpose in 2 of these studies and with a monitoring assignment in the other 2 studies. The content of the text messages and other monitoring aspects was directly linked to the various surgeries performed (Table 2). Four (36%) studies examined different outcomes after knee or hip surgery, two each following tonsillectomy or fracture (18%), and one each after a C-section, hand surgery, or colorectal surgery (9%) (Table 1).

**Table 2:** Overview of the interventions.

Study	Message purpose	Time (postoperative days)	Opioids	Pain scale	Alerts	Reminders
Anthony et al [32]	Monitoring	7	yes^a^	0-10	no	yes
Anthony et al [33]	Monitoring	14	yes	0-10	no	no
Booth et al [36]	Monitoring	60	yes	0-10	yes	no
Brix et al [35]	Adherence/monitoring	4	no	0-10	yes	no
Carrier et al [37]	Monitoring	7	yes^a^	0-10	no	yes
Chen et al [38]	Monitoring	14	yes^a^	0-10	no	yes
Day et al [39]	Education/adherence	14	yes^a^	no	yes	no
Nelson et al [40]	Monitoring	21	yes	0-10	no	no
Newton and Sulman [41]	Education/adherence	9	yes^a^	no	yes	no
Premkumar et al [42]	Monitoring	42	yes^a^	0-10	no	no
Yahanda et al [34]	Adherence/monitoring	15	yes^a^	0-9	no	yes

^a^No specific information about the type of opioid used given.

### Process of the Interventions

Whether the goal of the intervention was for patient monitoring or to analyze the adherence to pain management, the studies defined different timeframes. With 60 intervention days, one study was distinctly longer than the others [36] and can therefore be considered an outlier. The average timeframe of the remaining 10 interventions was 14.7 days for postoperative care. The individual outcomes were determined by various message blocks consisting of several SMS messages sent on different postoperative days. Eight of the 11 (73%) interventions transmitted at least one of these blocks daily, 1 study sent a message every second day [37], and 2 studies each sent messages at a certain time interval [40,41]. The first intervention informed patients daily in the first week, and then on the 10th, 14th, and 21st postoperative day [40]. The other intervention sent messages daily for the first 3 postoperative days, and then on the 5th, followed by the 7th to 9th postoperative days daily [41]. Within these time intervals, six interventions measured the outcome with one message block, usually in the morning. Two interventions sent one message block each morning and evening to the patients [40,42]. Three interventions sent an additional block at 12 PM [32,33,35]. Each of the blocks consisted of at least one question sent by SMS. In 7 of the 11 (64%) interventions, one block was subdivided into at least three SMS.

For 7 of the 11 (64%) studies, the message blocks’ primary outcome was the monitoring of the postsurgical pain level of the investigated patients. However, 3 of the 11 (27%) studies focused primarily on adherence to the treatment [34,35,41], and 1 (9%) study focused on the satisfaction level of the patients [39]. Further secondary results included drug intake, patient satisfaction, or the number of alert messages, among others. In total, 9 of the 11 (81%) studies measured current pain levels, either as a primary or as a secondary outcome. For this purpose, 8 of these studies used a pain scale from 0 to 10, and one used a scale from 0 to 9 [34]. Response 0 always reflects little or no pain, and 9 or 10 indicates the most severe pain. Three interventions focused on postoperative pain management for children, thereby involving legal guardians in the process [38,40,41]. For this monitoring, two studies utilized Wong-Baker Faces [38,40], which is a valid and effective method of assessing pain in children [43]. Postoperatively, patient groups received opioids in 10 interventions. Percocet [33], oxycodone [33,40], morphine [33,36], or fentanyl [36] was prescribed depending on the intensity of pain and severity of the procedure (Table 2).

Four out of the 11 (36%) interventions sent additional reminders to patients, and 4 interventions (36%) also sent alerts to patients and physicians (Table 2). Patients were reminded when they missed answering questions for several days [36], and one intervention provided three daily reminders regarding medication intake [35]. Alerts were triggered whenever various thresholds or schedules were exceeded, or when communication with the system occurred at an unscheduled time [32,34,37,38]. Once alerted, health care professionals organized the appropriate actions, initially by contacting the patients directly. Depending on these alerts, reminders, current symptoms, and responses to the respective message blocks, the pain management could be adjusted. For instance, alerts could lead to a change in medication or a follow-up examination.

### Intervention Results of Selected Studies

All studies identified a positive effect of SMS on pain management, thereby indirectly providing various recommendations for action. First, 7 interventions measured response rates, each with concrete results. Overall, between 8 and approximately 400 messages were sent to patients or legal guardians. With an average response rate of approximately 89.5% (SD 3.8%), the reliability of SMS as a communication and monitoring tool is evident, especially in comparison to conventional communication methods (Table 3). One study referred to two interventions where the response rate was 63% for telephone calls and 72% for a mobile app [36]. The response rate via SMS was significantly higher than that for email interventions (66.63%, *P*<.001) [42]. However, the researchers were unable to establish an association between response rate and age, level of education, and working status of the patients.

Further, the response rate was confirmed to decrease steadily over the intervention duration, and the majority of unanswered messages occurred within the last postoperative days [33,37,38]. The highest pain levels were measured in the first postoperative days [32,33,38,40] and then decreased daily to clinically unimportant levels [38,40]. One study showed that postoperative opioid use had a strong positive correlation with the reported pain (*r*=0.972, *P*<.001) [40]. All interventions that assessed medication intake confirmed this trend [32,33]. Six studies were verified and approved by respective institutional review boards (IRBs). Utilizing SMS for data collection was further deemed to be compliant with the Health Insurance Portability and Accountability Act (HIPAA) in three studies [32,33,40] (Table 3).

**Table 3:** Summary of systematic literature review results.

Reference	Outcome	Results	SMS messages sent, N	Response rate (%)	Compliance
Anthony et al [32]	Effective	Highest pain level within first 48 h; average use of 15.9 prescription opioids	~19	88.3	IRB^a^/HIPAA^b^
Anthony et al [33]	Effective	Response rate, pain, and medication intake decline over time	~22	87.5	IRB/HIPAA
Booth et al [36]	Positive impact	Rate especially powerful compared to traditional methods	~400	82.0	IRB
Brix et al [35]	Positive and efficient	Nonsignificant trend for better adherence	~8	n/a^c^	n/a
Carrier et al [37]	Positive impact	Intervention led to earlier detection	16	89.5	n/a
Chen et al [38]	Positive impact	Real-time monitoring possible	14	88.0	n/a
Day et al [39]	Positive impact	High satisfaction rate, high adherence and acceptance among patients	~18	n/a	IRB
Nelson et al [40]	Positive impact	Less medication intake, pain decreased daily	~20	88.4	IRB/HIPAA
Newton and Sulman [41]	Positive impact	Improved adherence and communication quality, less anxiety, positive educational effect	12	n/a	n/a
Premkumar et al [42]	Positive impact	Real-time, highly accepted, and available data collection method	~80	96.1	n/a
Yahanda et al [34]	Positive impact	Improved adherence and satisfaction	~18	n/a	IRB

^a^IRB: institutional review board.

^b^HIPAA: Health Insurance Portability and Accountability Act.

^c^n/a: not available; the article does not provide corresponding information.

## Discussion

### Principal Results

SMS-based pain management is highly applicable and efficient for postoperative communication between health care professionals and patients or legal guardians. Furthermore, alarms and reminders via SMS can improve and maintain communication, while supporting patients or their legal guardians. This support function is desirable for effective pain management [44]. Three of the 11 (27%) interventions even preoperatively communicated with patients [34,39] or their legal guardians [41]. This preoperative communication via SMS yields equally positive results as postoperative pain management; various studies confirmed this conclusion. Patients appear to be satisfied with preoperative preparation before treatment [45], while the legal guardians considered an automated SMS system as a beneficial support system [46]. One study utilized days 7, 4, 2, and 1 [39], whereas another used days 14, 4, 2, and 1 before surgery [41] for the intervention. Both studies relied on SMS for providing additional information regarding the surgery and specific process steps. Moreover, the text messages functioned as reminders to adhere to schedules and specific requirements or prerequisites of the intervention. The third study used two preoperative SMS messages to ensure that the prescribed medication was purchased and used correctly by the patient, starting 6 days before surgery [34]. Accordingly, 10 interventions averaged 17.4 days for pre- and postoperative care, and 14.7 days for purely postoperative care.

SMS technology was associated with positive results for all studies. The ubiquity of SMS makes it a cost-effective and straightforward method for pain management that is valid and less intrusive [35,38,40,42]. In addition to the very high response rate, the patients also responded quickly. One study determined an average response time of fewer than 12 minutes [37], further demonstrating the increased acceptance and utilization of SMS. This could enable the critical drivers of the opioid epidemic, such as inadequate medical education and guidelines, to be addressed directly, and most likely very effectively and efficiently. Nine interventions successfully monitored pharmaceutical opioids, and individually adjusted the pain management according to pain perception and response to the respective message blocks. The SMS systems improved adherence to pain management, and one study even reported less medication intake overall [40]. Patients could be reliably contacted, facilitating valuable information, education, and further questions. Therefore, an implementation of SMS-based pain management could combat the opioid crisis. Toward this end, process automation could be a crucial aspect. Automated SMS systems for pain management enable more robust data collection without consuming limited health resources, especially regarding personnel.

Furthermore, the use of SMS can prevent potential bias, and ensure the consistency and timeliness of messaging to patients [32,33,35,37,42]. Automatic postoperative communication has already been shown to reduce opioid intake in patients with orthopedic trauma [47]. One study also emphasized that automated alerts have enabled the more efficient and effective detection of postoperative complications [37]. These included, among others, pain above a certain level, no responses, missing acquisition of medications, and specific symptoms [34,37]. The information elicited through SMS had a net benefit in fewer telephone calls, saving time and personnel costs [39]. Generally, the constant messages and communication led to a positive patient experience. SMS improved the patients’ understanding and responsibility, and reduced their anxiety regarding the operation [39,41].

SMS-based pain management allows for simple pre- and postoperative extensions such as easily integrable and more specific questions about the operation and possible symptoms. In addition, reminders and alerts can be triggered automatically by SMS systems. Nevertheless, the response rate was confirmed to decrease steadily over the intervention duration, and the majority of unanswered SMS messages occurred within the last postoperative days [33,37,38]. Therefore, the extensions should be limited and the pain management period should be as short as possible, depending on the operation. The development of pain intensity and medication intake during each intervention supports this assumption. In conclusion, depending on the operation and the associated pain intensity, a monitoring timeframe and a medication schedule should be defined.

### Limitations

This study is subject to various limitations. First, only a basic quality analysis of the identified studies was applied. A more detailed analysis could clarify whether the final pool is rigorous, relevant, and credible. However, demand for high-quality research approaches such as randomized control studies is identified, as only one study was a nonblinded randomized control trial [35]. Second, the utilized Boolean search string could be defined more precisely. For instance, the results indicated that preoperative pain management has a decisive influence on an intervention’s success. Therefore, keywords such as “preoperative care*” or “presurgical care*” should be added. By enriching the search string, a more accurate result could be obtained.

### Conclusions

This study provides a comprehensive review of the current status of the literature on postoperative pain management by SMS. SMS utilization as a communication channel appeared to be favorable and feasible in pain management in the postoperative phase. According to three studies, SMS also seems to be useful for preoperative pain management, especially for additional information on medication or schedules, or as reminders. SMS resulted in excellent patient response rates, better adherence to pain treatment, higher patient satisfaction, and less medication intake. Six SMS interventions were certified by the respective IRBs and three were HIPAA-compliant. This indicates that SMS is capable of meeting health care requirements and is suitable for a health care–specific application. All of these benefits could be leveraged to address the opioid epidemic directly, effectively, and efficiently. The ability to create efficient pain management via SMS ensures comprehensive monitoring and communication. Key drivers of the opioid epidemic, such as medication abuse, shifted patient expectations, inadequate medical education, or inadequate guidelines, could be adequately addressed. In conclusion, the research question could be confirmed: SMS is effective, very well-suited, and feasible for postoperative pain management.

Future research should extend this systematic literature review regarding preoperative pain management. Based on this, a pre- and postoperative communication model should be developed to address the opioid epidemic effectively. This model should be generally applicable and adaptable to the individual clinical situation.

## Appendix

Multimedia Appendix 1 Systematic review protocol.

## Abbreviations

HIPAA: Health Insurance Portability and Accountability Act
IRB: institutional review board
