# Supplementary material for: Efficacy of Short Message Service Text Messaging Interventions for Postoperative Pain Management: Systematic Review
Source: JMIR Mhealth Uhealth. 2021 Jun 16;9(6):e20199. doi: 10.2196/20199 (PMC8277313; doi:10.2196/20199)
Supplement: Multimedia Appendix 1 [file mhealth_v9i6e20199_app1.pdf]

The Efficacy of Short Message Service for Postoperative Pain Management: Systematic Review Protocol

Christoph Buck[1], MSc, PhD; Christian Keweloh[2], BA, BSc; Adam Bouras[c], MHA; Eduardo J. Simoes[d], MD, MSc, MPH

[a] Queensland University of Technology, School of Management
[b] University of Bayreuth, Chair of Information Systems Management
[c] University of Missouri, School of Medicine
[d] University of Missouri, School of Medicine

Corresponding Author:
Christoph Buck
School of Management
Queensland University of Technology
2 George St, Brisbane City QLD 4000
Phone: +61405855937
Email: christoph.buck@qut.edu.au

Background

In the United States, more than 700,000 citizens died from drug overdoses between 1999 and 2017, about two-thirds of them due to opioids [1,2]. Addiction to opioids, especially to pharmaceutical opioids, poses a major public health threat [3,4]. These pharmaceutical opioids are typically prescribed for post-operative pain management [5]. According to the World Drug Report of the United Nations Office on Drugs and Crime 2019 (UNODC) [4], appropriate pain management strategies must be developed and implemented to respond adequately to this threat. These pain management strategies particularly rely on effective physician-patient communication [6]. With advancing digitalization, however, this communication has to be increasingly provided through telemedicine [7]. According to Lu et al. [8], mobile instant messaging applications (MIM), as well as short message services (SMS) offer major potential, whereby SMS is more frequently utilized. SMS communication provides a variety of features and proven benefits for healthcare applications [9,10]. To address this opioid epidemic, pain management combined with SMS as a communication medium could be viable, whereby the postoperative phase seems to be particularly decisive. Therefore, this paper aims to structure the current state of the literature regarding postoperative pain management via SMS. To our knowledge, there is currently no specific literature review on SMS-based pain management and no synthesized results.

Objectives

By answering the research question through a systematic literature review, a conceptual framework for future research should be provided. This paper examines the following research question:
Is postoperative pain management feasible and effective utilizing SMS?

Methods

The systematic literature review will be based on the recommendations of several guidelines [11–13].

Exclusion & Inclusion Criteria

Type of Publication:

Only studies that have been published in a scientific journal or at a conference will be included in the systematic literature review.

Language:

Only studies that have been published in English will be included in the systematic literature review.

Duplicates:

All duplicates will be excluded.

Type of Intervention & Outcome:

All studies that deviate thematically after conducting the Title-Abstract-, or Full-Text-Screening, will be excluded.

Research Method:

All studies with a secondary research method will be excluded.

Based on the remaining studies, a forward and backward search will be executed.

Search Strategy

The search string is designed by using Boolean operators. The search string will be adapted to the specific characteristics and requirements of the respective database. The following databases will be used for the literature search: PubMed, Medline, Scopus, Web of Science, CINAHL, Science Direct, IEEE, and AISeL.

"text messag*" OR "short messag*" OR "sms"
AND
"postoperative care*" OR "post-operative care*" OR "surge*" OR "surgic*" OR "operat*"
AND
"pain" OR "medicat*" OR "opioid*" OR "analgesic"

Data Collection & Data Synthesis

The relevant studies will be grouped and described according to study design, research objective, surgical procedure, automation, message purpose, time, pain scale and other aspects as appropriate.
The results of the systematic literature review will serve as a basis for developing a communication model.

Data Display

The results will be displayed by the following systematic literature review flowchart [13,14].

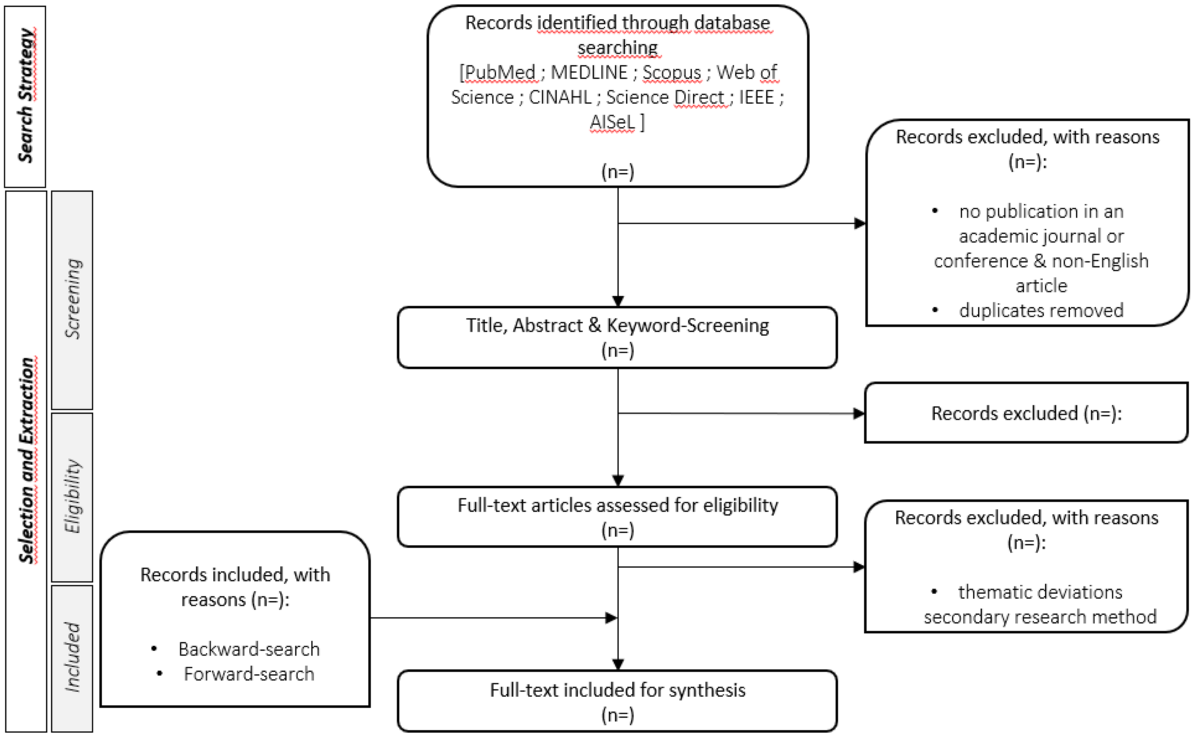

Additionally, the key results are summarized and presented in tables as outlined below.

**Study**	**Study Design**	**Research Aim**	**Surgical Procedure**	**Autom ation**	**...**
Study 1					
Study 2					
...					

Results

The systematic literature review process will be conducted between March and April 2019. The results will be consolidated and evaluated from April 2019.

References

1. Hedegaard H, Miniño AM, Warner M. Drug Overdose Deaths in the United States, 1999-2017. NCHS Data Brief 2018(329):1-8. PMID:30500323
2. CDC. 2018 Annual Surveillance Report of Drug-Related Risks and Outcomes - United States: Surveillance Special Report: Centers for Disease Control and Prevention; 2018.
3. Fischer B, Keates A, Bühringer G, Reimer J, Rehm J. Non-medical use of prescription opioids and prescription opioid-related harms: why so markedly higher in North America compared to the rest of the world? Addiction 2014;109(2):177-181. PMID:23692335
4. UNODC. World Drug Report 2019: (Set of 5 Booklets). [S.l.]: UNITED NATIONS; 2019. ISBN:978-92-1-148314-7.
5. Volkow ND, McLellan TA, Cotto JH, Karithanom M, Weiss SRB. Characteristics of opioid prescriptions in 2009. JAMA 2011;305(13):1299-1301. PMID:21467282
6. Henry SG, Matthias MS. Patient-Clinician Communication About Pain: A Conceptual Model and Narrative Review. Pain Med 2018;19(11):2154-2165. PMID:29401356
7. Haggerty E. Healthcare and digital transformation. Network Security 2017;2017(8):7-11. doi:10.1016/S1353-4858(17)30081-8
8. Lu K, Marino NE, Russell D, Singareddy A, Zhang D, Hardi A, Kaar S, Puri V. Use of Short Message Service and Smartphone Applications in the Management of Surgical Patients: A Systematic Review. Telemedicine Journal & E Health 2018;24(6):406-414. PMID:29111887
9. Miloh T, Annunziato R, Arnon R, Warshaw J, Parkar S, Suchy FJ, Iyer K, Kerkar N. Improved adherence and outcomes for pediatric liver transplant recipients by using text messaging. Pediatrics 2009;124(5):e844-50. PMID:19822583
10. Smith A, Rainie L, McGeeney K, Keeter S, Duggan M. U.S. Smartphone Use in 2015 URL: https://www.pewresearch.org/internet/2015/04/01/us-smartphone-use-in-2015/ [accessed 2020-12-07].
11. Kitchenham B. Guidelines for performing Systematic Literature Reviews in software engineering: EBSE Technical Report; 2007.
12. Okoli C, Schabram K. A Guide to Conducting a Systematic Literature Review of Information Systems Research. SSRN Journal 2010. doi:10.2139/ssrn.1954824
13. Moher D, Liberati A, Tetzlaff J, Altman DG. Preferred reporting items for systematic reviews and meta-analyses: the PRISMA statement. PLoS Med 2009;6(7):e1000097. PMID:19621072
14. Thuan NH, Antunes P, Johnstone D. Factors influencing the decision to crowdsource: A systematic literature review. Inf Syst Front 2016;18(1):47-68. doi:10.1007/s10796-015-9578-x
